# P-1416. Preference for Long-acting Injectable Tuberculosis Preventive Treatment over Oral TB Preventive Therapy among Healthcare Workers

**DOI:** 10.1093/ofid/ofaf695.1603

**Published:** 2026-01-11

**Authors:** Victoria Ontiveros, Zaza Avaliani, Luka Khelaia, Papuna Papuashvili, Ruth Demissie, Dzigbordi Kamasa-Quashie, A Nichole Evans, Angie Campbell, Naira Chanukvadze, Nelly Solomonia, Natalia Tsereteli, Tekla Madzgharashvili, Lauren F Collins, Russell R Kempker, Maia Kipiani, Matthew J Magee

**Affiliations:** Emory University Rollins School of Public Health, Atlanta, Georgia; National Center for Tuberculosis and Lung Diseases, Tbilisi, T'bilisi, Georgia; National Center for Tuberculosis and Lung Diseases, Tbilisi, T'bilisi, Georgia; National Center for Tuberculosis and Lung Diseases, Tbilisi, T'bilisi, Georgia; Emory University Rollins School of Public Health, Atlanta, Georgia; Emory University Rollins School of Public Health, Atlanta, Georgia; Emory University Rollins School of Public Health, Atlanta, Georgia; Rollins School of Public Health, Atlanta, Georgia; Salih Abashidze Regional Center of Infectious Pathology, Aids and Tuberculosis, Batumi, Ajaria, Georgia; National Center for Tuberculosis and Lung Diseases, Tbilisi, T'bilisi, Georgia; National Center for Tuberculosis and Lung Diseases, Tbilisi, T'bilisi, Georgia; National Center for Tuberculosis and Lung Diseases, Tbilisi, T'bilisi, Georgia; Emory University School of Medicine, Division of Infectious Diseases, Atlanta, Georgia; Emory University School of Medicine, Atlanta, GA; National Center for Tuberculosis and Lung Diseases, Tbilisi, T'bilisi, Georgia; Emory University, Atlanta, Georgia

## Abstract

**Background:**

Suboptimal adherence to daily/weekly oral tuberculosis preventive treatment (TPT) regimens among tuberculosis (TB) contacts poses a challenge to TB infection management. Long-acting injectable (LAI) formulations of TPT are in development and may help overcome some of the barriers to oral TPT. We assessed the acceptability of LAI TPT among healthcare workers (HCWs) in a high burden setting.

**Methods:**

We surveyed physicians and nurses at TB clinics in the country of Georgia from June–July 2024 using a Unitaid 40-question survey. Questions explored overall preference for LAI versus oral TPT, preferences for frequency of injection administration, and attitudes toward LAI TPT. We compared HCWs’ overall preference for LAI TPT by HCW characteristics and opinions of TPT formulation using prevalence ratios (PR) and 95% confidence intervals (CI).

**Results:**

Among 127 HCWs, median age was 57 years (interquartile range: 47–63), 86% were female, and 59% were physicians. Overall, 52% favored using LAI versus oral TPT and 36% remained unsure. Further, 66% were in favor of a 1-injection LAI regimen over oral therapy; 65% were in favor of a 2-injection LAI regimen (1x/month for 2 months); 63% were in favor of a 3-injection regimen (1x/month for 3 months). Preference for LAI versus oral TPT did not differ by age group or occupation. Male HCWs were non-significantly more likely to prefer LAI compared to female HCWs (PR 1.3, 95% CI 0.9, 2.0). HCWs who believed that oral TPT is effective (versus did not believe) were non-significantly more likely to prefer LAI (PR 1.4, 95% CI 0.7–2.6). The belief that TPT should be a priority of the National TB Program (versus should not be a priority) was non-significantly associated with being in favor of LAI TPT (PR 1.3, 95% CI 0.7–2.6). HCWs’ primary concerns regarding LAI were the potential for long-lasting and unexpected side effects, need for multiple injections, and lower efficacy. HCWs reported that if patients had severe liver disease, chronic comorbidities, or struggled to adhere to oral regimens, they would be more likely to recommend LAI over oral TPT.

**Conclusion:**

There was high acceptability for LAI TPT among doctors and nurses in a high burden setting. There may be potential to increase acceptability if LAI are as efficacious and safe as oral TPT regimens.

**Disclosures:**

All Authors: No reported disclosures

